# Optimising same day discharge hemithyroidectomy: defining outcomes, unplanned admissions and patient experience

**DOI:** 10.1308/rcsann.2025.0049

**Published:** 2025-07-15

**Authors:** W MacFaul, M Wojtowicz, B Puttergill, A McLaren

**Affiliations:** Stoke Mandeville Hospital, UK

**Keywords:** Day-case surgery, Hemithyroidectomy, Unplanned admissions

## Abstract

**Background:**

There is increasing emphasis on day-case hemithyroidectomy in the UK, with the national NHS/Get-it-right-first-time (GIRFT) recommending 30% zero-night stay rate. Buckinghamshire Healthcare Trust currently achieves >70% zero-night stay rate. This study profiles successfully treated day-case patients, evaluates reasons for unplanned admissions, complication rates and patient satisfaction, offering insights for units seeking to expand day-case practice.

**Methods:**

This is a retrospective cohort review from 2020 to 2024 of patients undergoing hemithyroidectomy treated by two surgeons: one with over a decade of day-case experience and one adopting the practice in 2021.

**Results:**

Of 336 patients (mean age 51±15 years, male:female 1:4), 283 (84%) were discharged on the same day with planned overnight admissions (37 patients, 11%) were primarily older (62 versus 50 years, *p*<0.05), more often American Society of Anesthesiologists (ASA) grade 3 (*p*=0.05), with a trend towards larger gland size, without correlation in either group to surgeon experience. Reasons for unplanned admissions in 14 patients (4%) were: anaesthetic concerns (*n*=3), intraoperative bleeding (*n*=2), large glands (*n*=2) and combined others, with all stays <48h. There was no correlation between unplanned admissions and patient age, ASA status or surgical indication. No hemithyroidectomy resulted in postoperative neck hemorrhage or return to theatre.

More than 80% of patients surveyed would choose same-day discharge hemithyroidectomy over an inpatient stay, with all of respondents reporting adequate pain control.

**Conclusions:**

With robust protocols and appropriate patient selection, high rates of day-case hemithyroidectomy are achievable, in alignment with a high rate of patient satisfaction. Unexpected admissions lack clear predictors.

## Introduction

Hemithyroidectomy has seen a growing trend towards day-case management (same day discharge) in appropriately selected patients, with Get it Right First Time (GIRFT) and the British Association of Day Surgery recently recommending a zero-night stay rate of 30%.^1^ Day-case surgery offers significant advantages, including reduced healthcare costs, high patient satisfaction and reduced hospital-acquired complications.^1,2^ However, despite these benefits, unexpected admissions following planned day-case hemithyroidectomy remain a concern; specifically, postoperative haemorrhage and airway complications have prevented a broader uptake of day-case hemithyroidectomy.^3,4^

The feasibility of day-case hemithyroidectomy is contingent on several factors, including meticulous patient selection, optimised perioperative care and robust discharge criteria.^5–7^ Rates of unplanned admissions vary widely across institutions, reflecting differences in clinical practices and the complexities of individual patient profiles.^1,8^ Common reasons for unexpected admissions include postoperative pain, nausea and vomiting, hypocalcemia, haematoma and patient preferences for extended monitoring.^6,9,10^ Understanding these factors is crucial for improving surgical pathways and enhancing the safety and efficiency of day-case hemithyroidectomy.

Our unit has been performing high rates of same-day discharge hemithyroidectomy for several years, with NHS model hospital demonstrating 70% attainment in 2023 (national benchmark 7.89%).

This study aimed to evaluate admission rates and contributing factors for hospital admission following planned day-case hemithyroidectomy in our healthcare service. ‘Day-case discharge’ refers to same-day discharge in our practice setting. We have further explored patient-related outcome measures for day-case hemithyroidectomy through patient survey. We hope to provide data to support the broader adoption of safe and effective day-case surgery models.

## Methods

A retrospective cohort study was completed on data from all patients who had undergone a hemithyroidectomy from January 2019 to October 2024. All cases were performed at the same hospital site, which has a standard operating procedure in place. Procedures were performed by two surgeons, one with over ten years of day-case hemithyroidectomy experience, one with three years of day-case hemithyroidectomy experience. We reviewed these data against model hospital data for readmissions outside of the trust.

Patient satisfaction surveys were conducted for patients who underwent hemithyroidectomy in the past six months.

### Day-case pathway

Patients were appropriately consented with information on day-case surgery provided in the outpatients clinic setting and again on the day of surgery. All day-case patients were admitted through the day surgery unit rather than an inpatient ward setting. Day-case surgery at our unit is performed at an elective hospital site, with access to emergency admission if required; Critical Care Outreach Team (CCOT) is present on site with training on neck haematoma management for CCOT and Intensive Care Unit (ICU).

The majority of patients underwent bilateral superficial cervical plexus blocks with local anaesthetic before start of surgery. All patients were observed for up to six hours following surgery and reviewed by a clinician before discharge. Safety netting card with ‘red flag’ symptoms for neck haematoma and instruction on return to hospital with contact details: Operating surgeon, Emergency services, Critical Outreach, On call Surgical Registrar.

The observation and discharge pathway ‘Standard Operating Procedure’ was approved at a trust level and is reviewed annually. Patients appropriate for day-case surgery had to satisfy appropriate selection criteria: hemithyroidectomy, no significant medical comorbidities, patients to be discharged home with appropriate support (for inclusion criteria, see Table 1). All patients are monitored for a minimum of four hours postprocedure.

**Table 1:** Inclusion criteria – all criteria must be met.

Day-case inclusion criteria
Hemithyroidectomy
Large goitre excluded
No significant comorbidities
Patients able to follow instructions
Patient to be discharged home with responsible adult for 24 hours
Surgeon decision pre-/intra-/postoperative consistent to support daycase

Criteria for discharge are: adequate analgesia and pain controlled, toileting, eating, no breathing difficulties, normal vitals, surgeon review of neck and wound, adequate support at home.

Patients are followed up in person as a routine outpatient’s appointment. Patients are excluded from day case if medical comorbidities assessed by surgical/anaesthesitic team determine inpatient monitoring; lobe size >100g is a relative contraindication to day-case pathway.

Patient anticoagulant use does not preclude day-case hemithyroidectomy. We use local trust guidance in regards to ceasing before surgery. For example, warfarin is stopped five days before surgery, Direct Oral Anticoagulants (e.g. Apixacan, Dabigatran) are ceased 48 hours before surgery and Clopidogrel is ceased 7 days before surgery.

### Data collection

Information collected included demographic information such as age and sex. Clinical information included: indication for surgery, ASA grade, admission status (planned day-case or planned overnight admission), successful day zero discharge or unexpected admission, reason for admission and gland size on resection specimen.

For patients who had undergone treatment in the six months before the end of the study period, a questionnaire was sent to determine patient experience of the day-case pathway with 30 patients surveyed.

### Data analysis

Descriptive data analysis and logistic regression was done in Excel (CI 95%, *p*=0.05).

## Results

A total of 336 patients underwent hemithyroidectomy between the two surgeons within the study period; descriptive data are presented in Table 2.

**Table 2:** Descriptive statistics

Variable	Measure
Age	51±15 years
Sex	
Male: female	1:4
Diagnosis (*n*)	
Diagnostic lobectomy	170
MNG	99
Malignancy	24
Completion Lobectomy	25
Cyst	14
Toxic adenoma	4
ASA	2 (average)

ASA = American Society of Anesthesiologists; MNG = multinodular goitre

### Successful day case

In all, 84% of patients (*n*=283) were managed successfully through the day-case pathway and discharged on day zero; 15% of patients (*n*=51) required admission, most admissions were planned admissions (*n*=37), with only 4% (*n*=14) of all patients requiring an unplanned admission.

The main indications for planned admission were gland size (*n*=13), followed by medical comorbidity (*n*=10), Table 3 lists all the indications for planned and unplanned admission.

**Table 3:** Comparison of characteristics between discharged and admitted patients

Variable	Discharged			Planned admission			Unplanned admission			*p*-value
Age (years)	50 (19–92)			62 (22–90)			52 (26–76)			<0.05
Gland (g)	19g (3.4–170g)			79g (10–227g)			46g (5.2–107g)			n/s
ASA (%)	1 (43%)	2 (52%)	3 (5%)	1 (20%)	2 (60%)	3 (20%)	1 (25%)	2 (66%)	3 (9%)	<0.05

ASA = American Society of Anesthesiologists

On regression analysis, patients planned for admission were more likely to be older (mean age 50 versus 62 years (*p*=0.002) and more likely to be ASA 3 (*p*=0.001). There was a trend towards larger gland size in those patients planned for admission, but this did not reach statistical significance. Of the 14 patients who required an unplanned admission, there was no obvious recurring cause (Table 4). All patients undergoing hemithyroidectomy were discharged within 48 hours, no patients required a return to theatre and there was no difference in admission rates between surgeon experience.

**Table 4:** Reasons for admission across planned and unplanned admissions

Reasons for planned admission	*N*=34	Reasons for unplanned admission	*N*=12
Gland size	13	Anaesthetic concern (difficult intubation/slow recovery/bradycardia)	4
Medically comorbid	10	Large gland	2
Combined other procedure	4	Postural hypotension	2
Reoperative	3	Late finish	2
BMI	2	Intraoperative bleeding	2
Anticoagulation	2	Pain	1
Social reasons	1	Postoperative arm weakness	1

ASA = American Society of Anesthesiologists; BMI = body mass index

### Patient feedback

**Figure 1:**
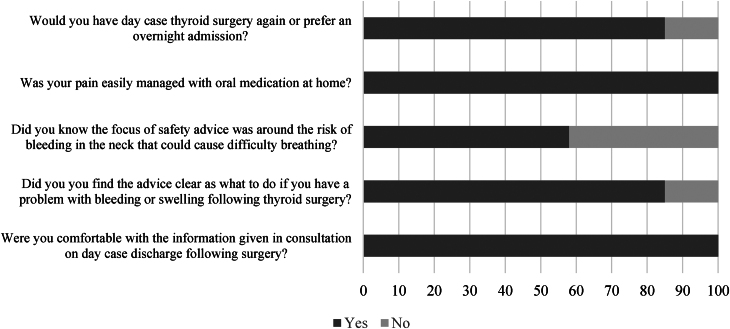
Patient survey for same day discharge following hemithyroidectomy

Patient response to the survey questions are detailed in Figure 1.

The responses demonstrate a high rate of satisfaction with same-day discharge following hemithyroidectomy having received adequate information and advice with all patients having adequate pain control. Free-text responses highlighted nuances in care that would enable a more comfortable recovery while in the surgical unit.

## Discussion

This study demonstrates that high rates of hemithyroidectomy can be performed as a day-case procedure, with no apparent increased risk. With robust standard operating procedure and discharge pathway, our day-zero discharge rate was >80%. Our results underscore the feasibility of achieving high rates of same-day discharge, even in a diverse patient cohort with varying indications for surgery and comorbidities. The findings align with the growing body of evidence supporting outpatient thyroid surgery, facilitated by advances in surgical techniques, anaesthesia and perioperative care.^6,9–11^

It was interesting to identify the level of satisfaction of pain control postoperatively – considering most patients are discharged with advice to use simple, over-the-counter analgesia, with opiate analgesia rarely prescribed on discharge. All patients managed through the day-case pathway who responded to the survey stated they had adequate pain control; future data exploring pain visual analogue scores between admitted patients undergoing thyroid surgery and day-case patients may reflect this more clearly, with increasing evidence from other surgical fields that patients who undergo outpatient surgery experience less pain than inpatients.^12,13^

Our day-case rates of >80% are higher than previously published UK series, of which the highest so far has been around 70%, with national rates of <10% quoted in UK Register of Endocrine and Thyroid Surgery (UKRETS) and GIRFT data.^1,8,9,10^ The success of day-case hemithyroidectomy in this series could be attributed to several factors. First, a robust preoperative selection process ensured that patients with significant comorbidities or anatomical challenges were more likely to undergo planned overnight admissions. Secondly, structured perioperative pathways with close postoperative monitoring and efficient discharge planning likely played a critical role.

### Planned admissions: gland size and patient age

Among the 15% of patients requiring overnight admission, 75% were planned. The primary drivers of planned admissions were anticipated challenges due to large gland size, combined second surgical procedure within the neck (on the same side), such as a parathyroidectomy, and patient comorbidities. Although we do not have a strict age cut off for day-case surgery, age was a statistically significant determining factor for planned admission, possibly related to elderly patients having higher burden of comorbidities and therefore higher ASA grade, impacting their recovery and suitability for day-case surgery. This is consistent with a retrospective study of >1,000 patients, whose findings were that those managed as day-case were younger and had a lower ASA grade.^6^ Our findings highlight the importance of preoperative planning and individualised risk stratification. For example, larger glands may pose a higher risk for prolonged operative times, increased intraoperative bleeding or postoperative swelling, necessitating extended observation. Interestingly, we were still able to discharge a number of elderly patients on day zero, highlighting the need for an individualised patient approach in some circumstances, and justifying our belief that age is not a contraindication to day-case surgery.

### Unplanned admissions: no clear predictors

Unexpected admissions occurred in 14 patients, which was 5% of those initially planned for discharge, with no clear pattern or correlation to patient demographics, ASA classification or surgical indication. The reasons were varied, with no single dominant factor, reflecting the inherent unpredictability of certain perioperative events. While intraoperative bleeding and anaesthetic concerns accounted for a portion of these cases, other reasons, such as late operating times and postoperative presyncope are logistical or temporary issues. A previous UK study also noted afternoon patients were more likely to be converted to admission due to reduced observation time, which is something important to consider when implementing day-case pathways.^10^ Although unpredictable events highlight the importance of provisions in the pathway for unexpected admissions, importantly, the lack of significant complications, such as reoperation for bleeding, and the short duration of stay (<48 hours) in these patients underscore the overall safety of the day-case approach.

### Patient experience of day-case surgery

Patients’ responses demonstrate that same-day discharge and safety advice is well received. Therefore, same-day discharge may well be aligned with patients’ preferences in postoperative care rather than the perception of cost reduction driving same-day discharge.

### Implications for clinical practice

These results provide valuable insights for optimising day-case thyroid surgery pathways. First, preoperative identification of factors such as gland size, combined procedures and comorbidities can guide the planning of overnight stays to prevent unplanned admissions. Second, efforts to minimise logistical issues, such as ensuring early operative start times, could further enhance the likelihood of day-zero discharge. Finally, while the low complication rate is reassuring, ongoing vigilance and monitoring protocols are essential to address any unforeseen postoperative events promptly. One of the factors limiting wider uptake of day-case hemithyroidectomy is concern regarding postoperative compressive haematoma. None of our patients required a return to theatre or bedside decompression of a haematoma; a recent large meta-analysis of >9,000 outpatient thyroidectomy patients had a postoperative bleed rate of 0.6%, with the authors concluding outpatient hemithyroidectomy was safe.^5^ Furthermore, a large retrospective cohort study of >1,000 patients investigating reoperation for bleeding after thyroid and parathyroid surgery showed only 1 out of 25 patients who bled did so in the 6–24 hour window, with 23 of 25 bleeding before 6 hours – further justifying the 6-hour observation period.^14^

### Limitations and future directions

This study is limited by its retrospective nature and the single-centre setting, which may limit generalisability. Additionally, the lack of a predictive model for unplanned admissions indicates the need for further research into potential risk factors that could enhance preoperative stratification. Prospective studies with larger cohorts could provide more robust data to refine day-case thyroid surgery protocols further.

Hemithyroidectomy performed with inpatient stay has a high patient satisfaction level; our patient survey, which we believe is the index UK data on Patient Related Outcome Measures in day-case thyroidectomy, shows that patients are also satisfied with day-case thyroid surgery. Other drivers of day-case lobectomy include reduction in hospital bed pressures, and reduction in care costs.

## Conclusion

Hemithyroidectomy can be performed as a day-case procedure in a high number of patients, with >80% of our patients discharged successfully on the same day. While planned admissions were effectively stratified based on gland size, age and comorbidities, unplanned admissions were rare, brief and without a predictable pattern. These findings support the expansion of day-case thyroid surgery, with continued emphasis on individualised preoperative planning and streamlined perioperative care pathways for those trusts interested in increasing their day-zero discharge rate.

## Ethics approval

This study was carried out under the Code of Ethics of the World Medical Association (Declaration of Helsinki). Approval was granted by the Ethics Committee/IRB of Buckinghamshire Healthcare Trust: Approval Reference: General Surgery/SE/2024-13/12.
